# Supplementary material for: Blockade of the SRC/STAT3/BCL-2 Signaling Axis Sustains the Cytotoxicity in Human Colorectal Cancer Cell Lines Induced by Dehydroxyhispolon Methyl Ether
Source: Biomedicines. 2023 Sep 13;11(9):2530. doi: 10.3390/biomedicines11092530 (PMC10526010; doi:10.3390/biomedicines11092530)
Supplement: Supplementary file 1 [file biomedicines-11-02530-s001.zip › biomedicines-2567987-supplementary.pdf]

Supplementary Figures for biomedicines-2567987 by Hsieh, Y.C. et al.

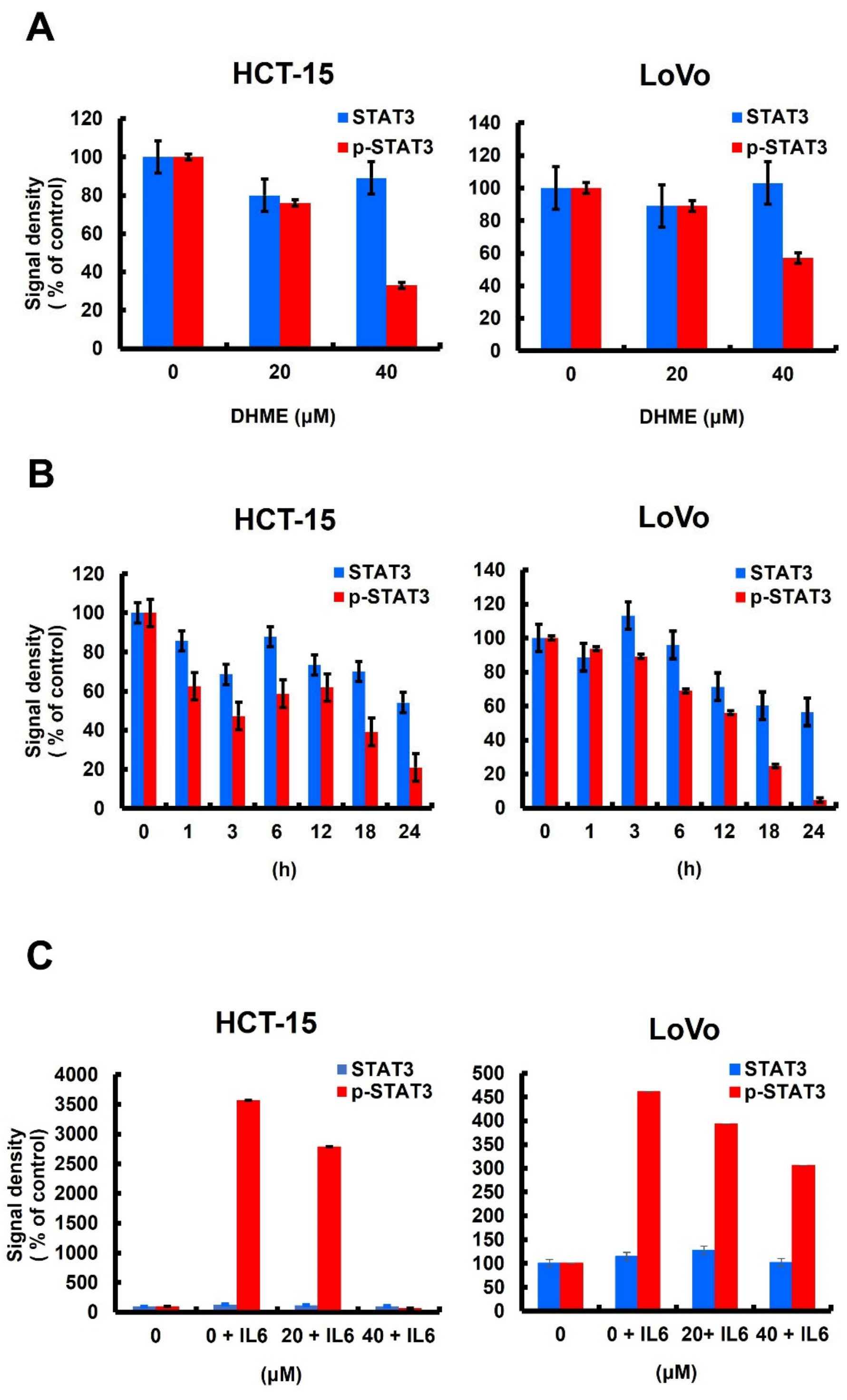

Figure S1. Quantitative results of the protein density shown in the Immunoblot images of Figure 2. (**A**) *Dose-dependent suppression of constitutive STAT3 activation by DHME.* (**B**) *Time-dependent suppression of constitutive STAT3 activation by DHME.* (**C**) *Suppression of IL-6-induced STAT3 activation by DHME.* In all immunoblot analyses, the amount of GAPDH was used as the control for equal loading. The levels of the protein-to-GAPDH ratio relative to drug-free controls on the blots were quantified using ImageJ algorithm.

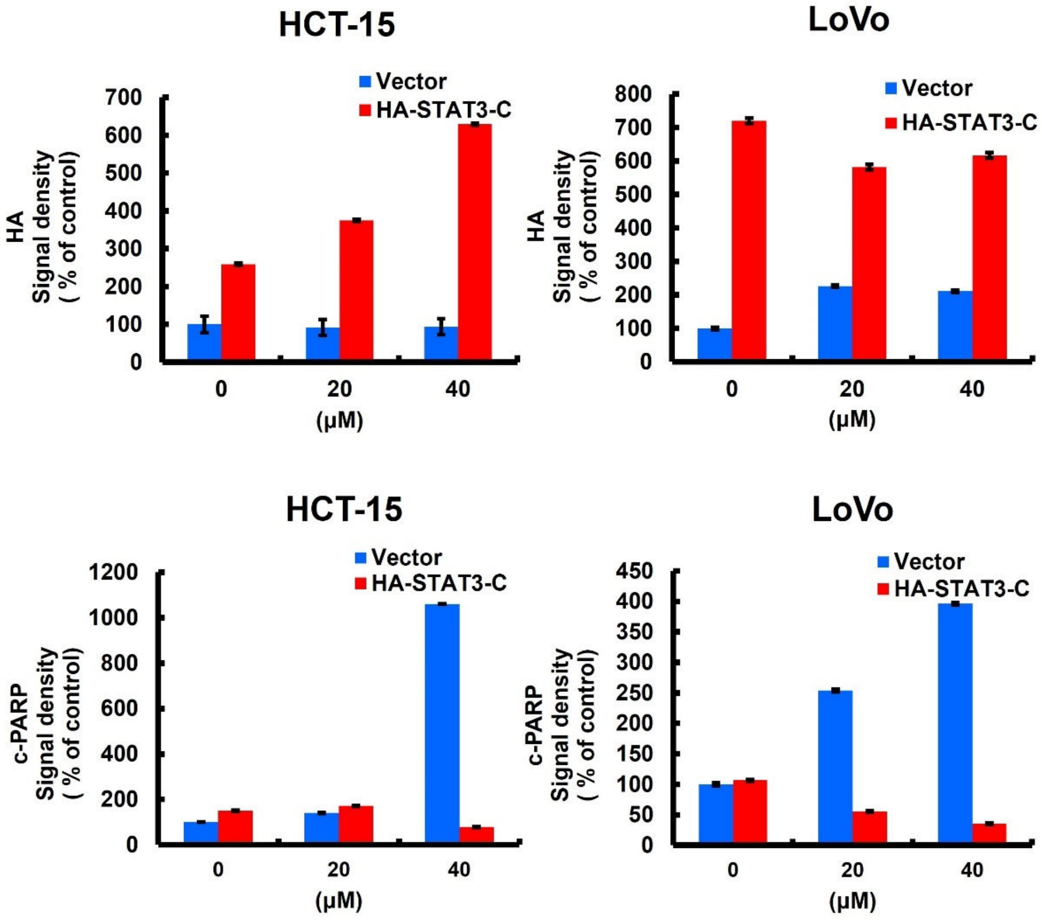

Figure S2. Quantitative results of the protein density shown in the Immunoblot images of Figure 3. *Sustained STAT3 activation blocks DHME-induced apoptosis.* Human CRC cell lines stably expressing vector alone or a dominant-active STAT3 mutant (STAT3 (A661C/N663C); STAT3-C) were treated with DHME (0, 20, 40 μM) for 24 h, followed by immunoblotting for the levels of cleaved PARP (c-PARP). GAPDH levels were used as equal loading control. ImageJ algorithm was used to quantify the levels of the protein-to-GAPDH ratio relative to drug-untreated controls and are indicated below each blot.

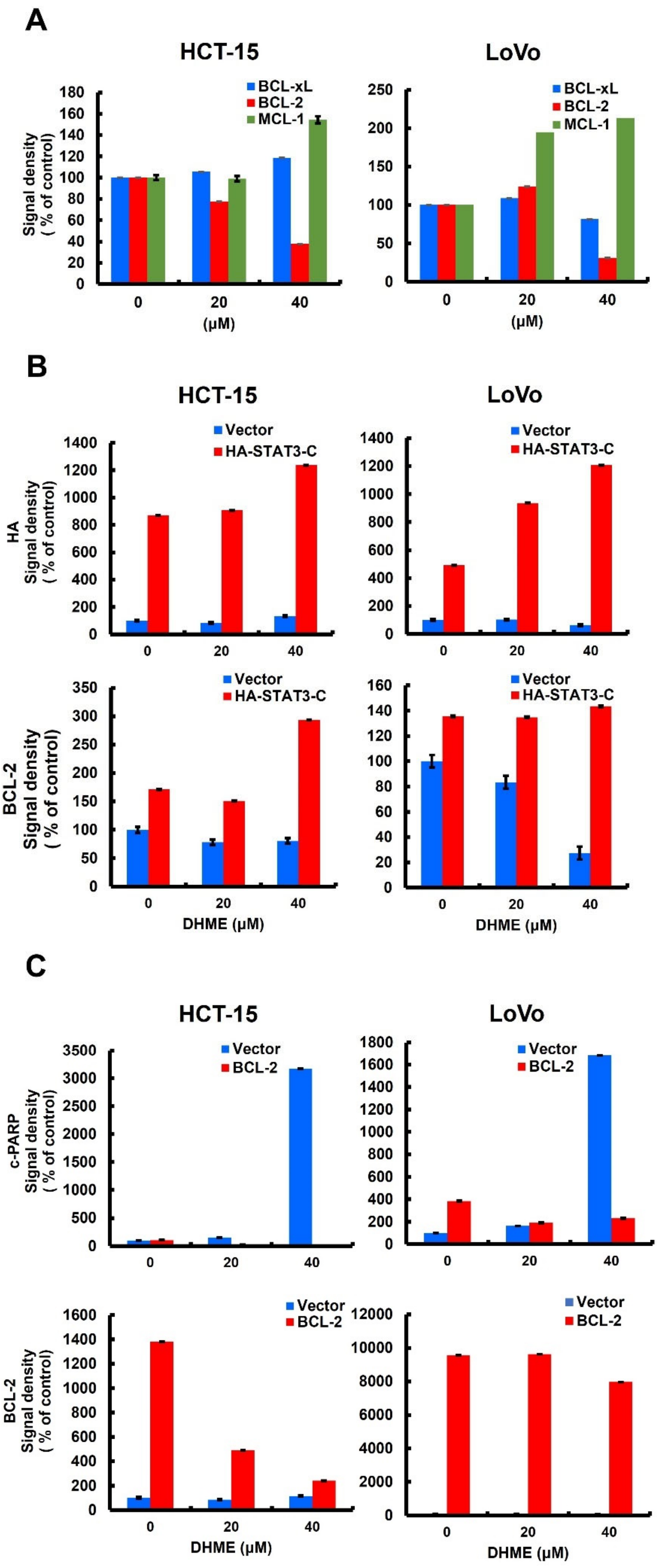
A
HCT-15
LoVo
BCL-xL
BCL-2
MCL-1
Signal density
(% of control)
(μM)
B
HCT-15
LoVo
Vector
HA-STAT3-C
HA
Signal density
(% of control)
BCL-2
Signal density
(% of control)
DHME (μM)
C
HCT-15
LoVo
Vector
BCL-2
c-PARP
Signal density
(% of control)
BCL-2
Signal density
(% of control)
DHME (μM)

Figure S3. Quantitative results of the protein density shown in the Immunoblot images of Figure 4. (**A**) *DHME downregulates BCL-2.* (**B**) *DHME downregulates BCL-2 in a STAT3-dependent manner.* (**C**) *DHME downregulates BCL-2 for inducing CRC cell apoptosis revealed by PARP cleavage (c-PARP)*. GAPDH levels serve as equal loading control. The levels of the protein-to-GAPDH ratio relative to drug-free controls were quantified using ImageJ algorithm and are denoted under each blot.

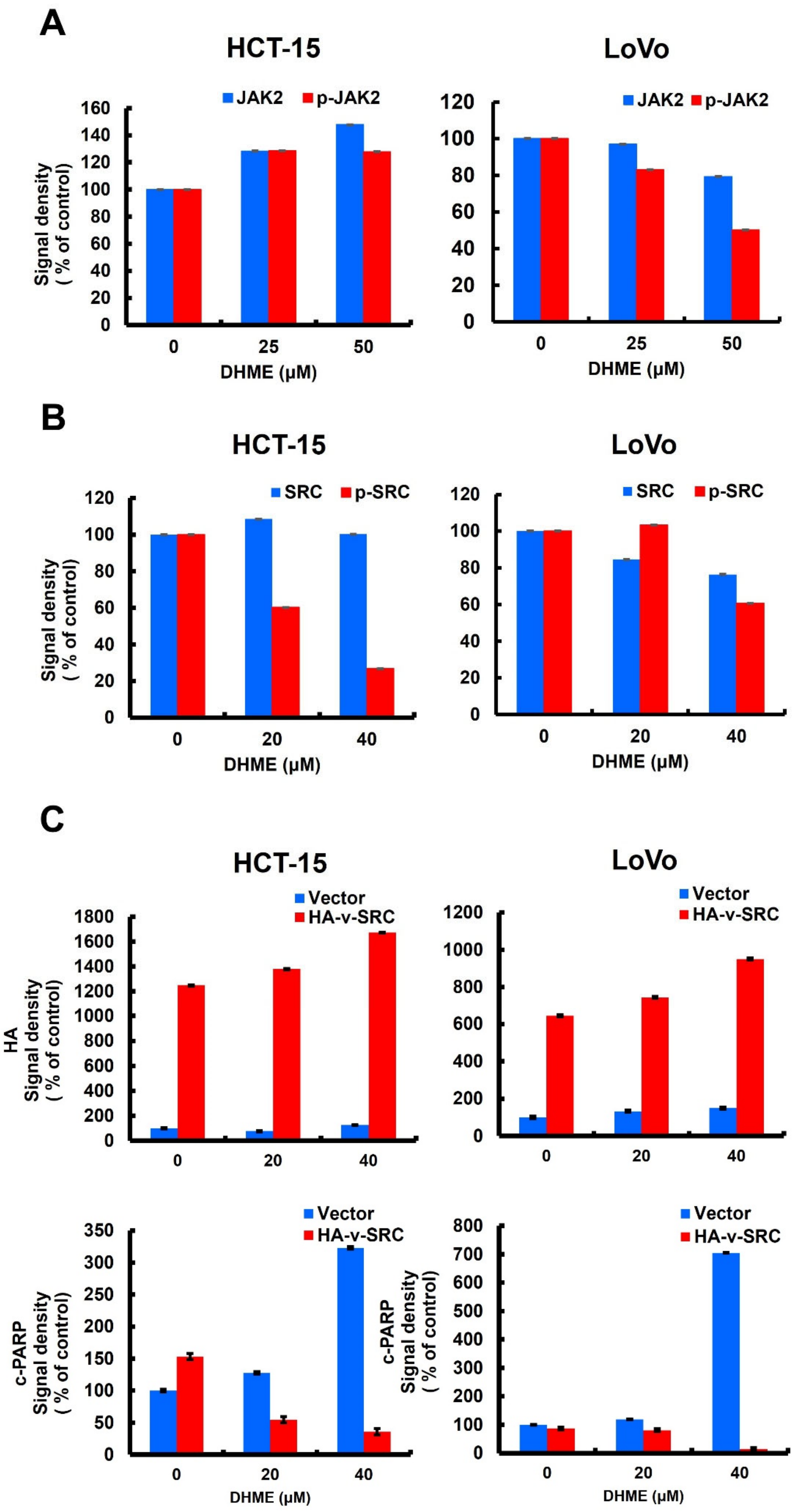
A
HCT-15
LoVo
JAK2
p-JAK2
Signal density
(% of control)
DHME (μM)
B
HCT-15
LoVo
SRC
p-SRC
Signal density
(% of control)
DHME (μM)
C
HCT-15
LoVo
Vector
HA-v-SRC
HA
Signal density
(% of control)
c-PARP
Signal density
(% of control)
DHME (μM)

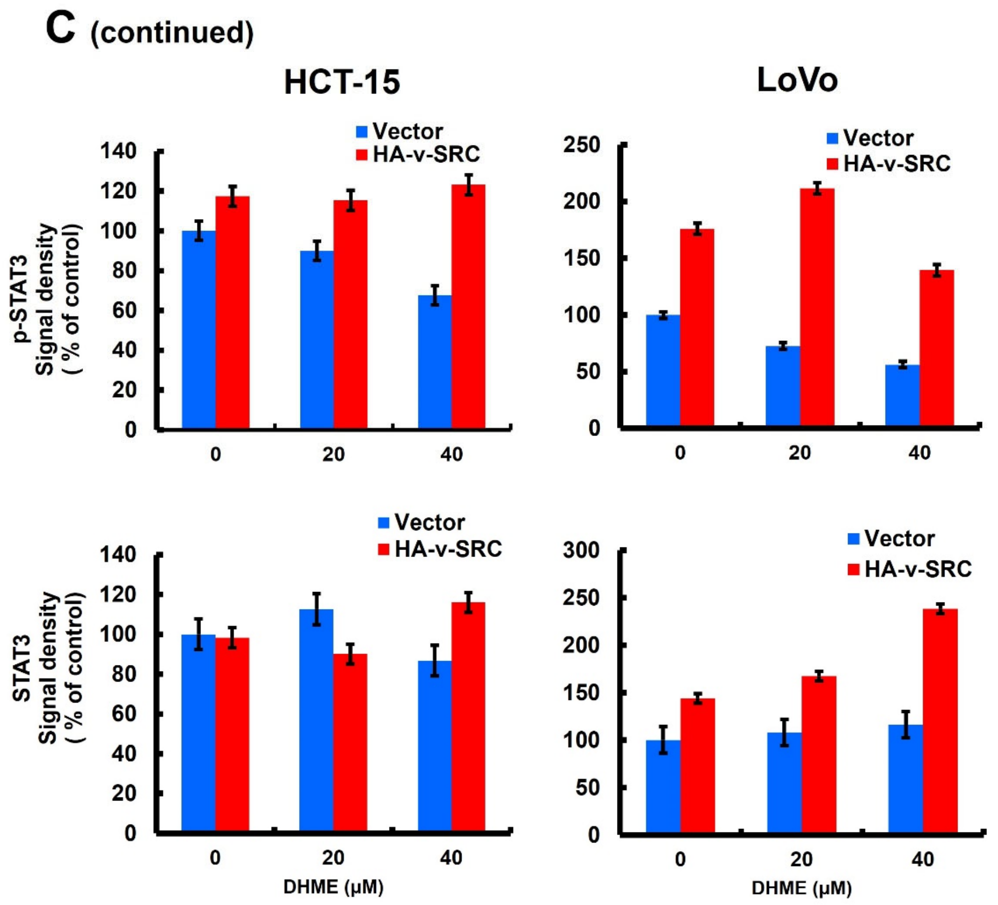

Figure S4. Quantitative results of the protein density shown in the Immunoblot images of Figure 5. (**A**) *Limited effect of DHME on JAK2 activation*. (**B**) *DHME suppresses the activation of SRC.* (**C**) *Sustained SRC activation thwarts DHME-induced blockade of STAT3 activation and apoptosis revealed by PARP cleavage (c-PARP).* The level of GAPDH was used as the control for equal loading. ImageJ algorithm was used to quantify the levels of the protein-to-GAPDH ratio relative to drug-free controls and are shown below each blot.
